# Spontaneous mirror symmetry breaking in heterocatalytically coupled enantioselective replicators†
†Electronic supplementary information (ESI) available: Methods and text on stability analysis. Five figures showing simulation examples. See DOI: 10.1039/c6sc02446g


**DOI:** 10.1039/c6sc02446g

**Published:** 2016-09-13

**Authors:** Josep M. Ribó, Joaquim Crusats, Zoubir El-Hachemi, Albert Moyano, David Hochberg

**Affiliations:** a Department of Organic Chemistry , University of Barcelona , E-08028 Barcelona , Catalonia , Spain . Email: jmribo@ub.edu; b Institute of Cosmos Science (IEEC-UB) , University of Barcelona , E-08028 Barcelona , Catalonia , Spain; c Department of Molecular Evolution , Centro de Astrobiología (CSIC-INTA) , E-28850 Torrejòn de Ardoz , Madrid , Spain . Email: hochbergd@cab.inta-csic.es

## Abstract

Chiral hypercycle replicators (first-order autocatalysis together with mutual cross-catalysis) formed from achiral or racemizing resources may lead to spontaneous mirror symmetry breaking (SMSB) without the need for additional heterochiral inhibition reactions, such as those of the Frank-like models, which are an obstacle for the emergence of evolutionary selection properties. The results indicate that the chemical models for the emergence of primordial autocatalytic self-reproducing systems, of and by themselves, can also explain naturally the emergence of biological homochirality.

## Abstract 2

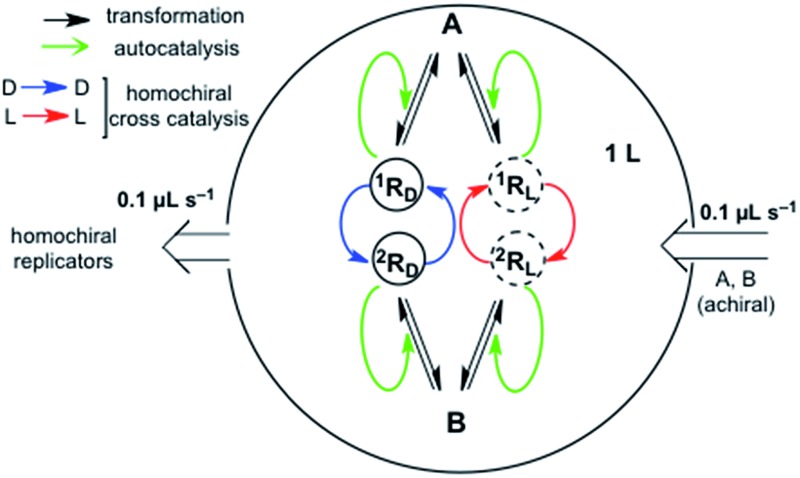
Hypercycles proposed as a chemical basis for the selection of biological replicators may lead to homochirality when fed from achiral resources.

## Introduction

The emergence of catalytic functionalities and autocatalytic systems form the basis for models of abiotic chemical evolution leading to self-reproducing systems: for example, autocatalytic sets,^1^ quasi-species^2^ and the RNA world.^3^ The coexistence, mutual stabilization and growth of self-reproducing chemical species are substantiated by models of sets of autocatalytic replicators,^4,5^ the seminal one being the hypercycle model consisting of replicator sets coupled together by mutual cross-catalysis.^6^ The chiral structure and enantioselective kinetics of actual autocatalytic replicator sets and of the RNA world are either entirely ignored altogether, or else simply assumed from the outset. This means that the emergence of biological homochirality^7,8^ is supposed to occur in processes either separated from, or in parallel with, the formation of the replicators. Furthermore, the emergence of biological homochirality is frequently considered to be merely an accidental singular process of chemical evolution, and not as a collective emergent phenomenon arising from the increase of complexity during chemical evolution. We show here how spontaneous mirror symmetry breaking (SMSB) emerges necessarily from hypercyclic dynamics when the chirality and enantioselectivity of the replicators are taken into account, and in conjunction with their formation from achiral resources. Common chemical transformations involve mono- and bimolecular reaction mechanisms, therefore autocatalysis is itself an uncommon transformation, and it is difficult to imagine how it could be higher than first order ([1]: quadratic non-linearity with respect to products/catalysts).

1 A + R ⇆ 2R

However, the growth rates leading to the survival of one replicator at the expense of another one must be greater than those provided by first-order autocatalysis [1].^9,10^ In the case of enantioselective autocatalysis (for A achiral and R chiral: R_D_ and R_L_ represent the replicator enantiomers),

2 A + R_D_ ⇆ 2R_D_      A + R_L_ ⇆ 2R_L_

then SMSB, *i.e.* the survival of only one of the two degenerate enantiomers,^8^ takes place only in low-order enantioselective autocatalysis when this is coupled to a heterochiral reaction: for example [3] such as in the Frank-like systems^11^ and [4] in the limited enantioselective reaction model.^12^

3 R_D_ + R_L_ → P_achiral_

4 R_D_ + R_L_ → A + R_D_      R_D_ + R_L_ → A + R_L_

Despite the dramatic experimental reports^13,14^ on SMSB, the models for this are not employed except as models for the evolution of self-reproducing species. This is because inhibition reactions, such as those in [3] and [4], present serious obstacles for the coexistence of self-reproducing chemical species. Furthermore, this has also prevented researchers from considering enantiomeric mixtures in replicator models, principally due to the experimental reports on strong heterochiral interactions (*i.e.* reactions such as [3]) in enantiomeric mixtures of oligonucleotides.^15^ However, a recent report on D- and L-RNA mixed systems shows that heterochiral inhibiting interactions occurring in RNA should not be taken as dogma.^16^ In this respect, it is certainly significant that the old report^15^ on heterochiral inhibitions in oligonucleotides refers to structures built by only one type of residue. Note that their tertiary structures, for example helices, are chiral but are of a higher order symmetry (or lower informational entropy) than those of actual biopolymers composed of different residues (with point group C_1_). This fact most likely hinders strong heterochiral interactions.

**Fig. 1:**
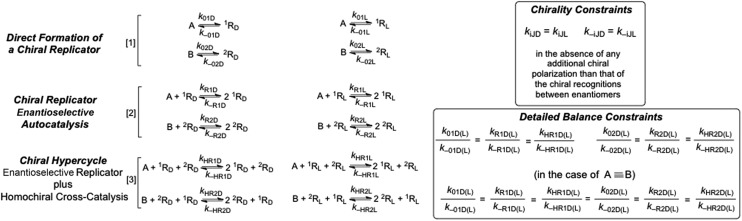
Transformations implied in the formation of two chiral replicators (^1^R_D_/^1^R_L_ and ^2^R_D_/^2^R_L_) from achiral or racemizing species (A and B) which by mutual cross-catalysis lead to a simple two replicator hypercycle.

We consider here the kinetics of chiral hypercycles, in various system architectures which maintain the reaction network out of thermodynamic equilibrium with its surroundings, that is, systems leading to non-thermodynamic final states as the most stable state of the system, as occurs in living biological systems^4^ and in systems leading to SMSB rather than the racemic mirror-symmetric mixture.^8^ In all the simulations only transformations (see Fig. 1) involving both the forward and backward reactions were considered. This allows one to describe a system obeying the constraints between the equilibrium constants and the reaction rate constants dictated by chemical thermodynamics^17^ and safeguards against obtaining purely artifactual SMSB results arising from violations of the second law of thermodynamics. In actual biological systems, replication is an autocatalytic transformation, but in abiotic chemical scenarios a previous direct synthesis of the replicators is clearly necessary. In fact, there are a few otherwise dramatic reports reporting the direct formation of self-replicating oligonucleotides and peptides.^18,19^ The simulations presented herein do not take into account how actual replicators may be obtained from achiral resources, nor what are the detailed reaction mechanisms underlying their autocatalytic processes (for example, template mechanisms), since our primary objective is to indicate how SMSB may occur in hypercyclic replicators.

## Results and discussion

The ability of cross-catalytically aided replicators to achieve a final dynamically stable chiral state (a non-thermodynamic state) instead of a racemic one, was simulated by deterministic kinetic rate equations (see the ESI†) in different systems that are maintained out of thermodynamic equilibrium with their surroundings. The systems so tested correspond to common chemical scenarios, which agree with the reductionist approach used in abiotic or proto-life scenarios.

### Systems closed to the replicator species and their resources but externally chemo-driven

**Fig. 2:**
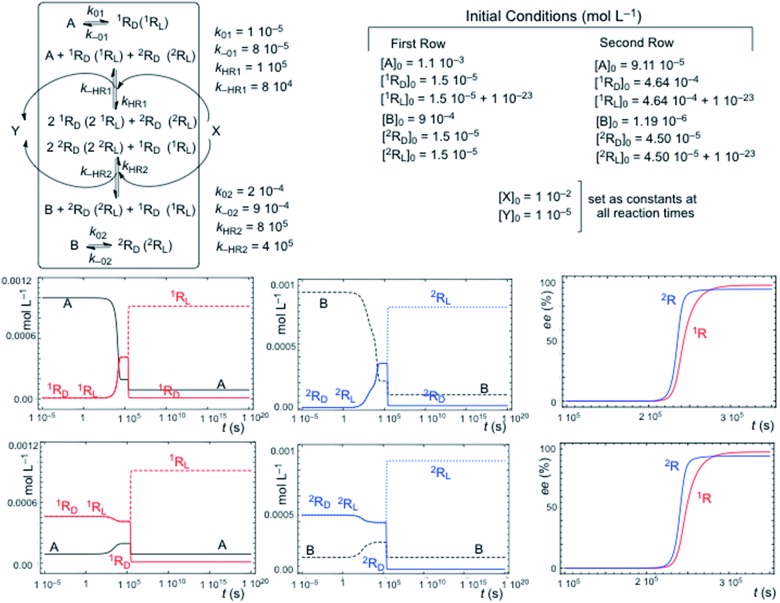
SMSB of the hypercycle reaction network [1] + [3] in a system open to the external reagent X/Y that drives the autocatalytic transformation. In this system, closed to the replicator species (A, B, ^1^R_D_ (^1^R_L_) and ^2^R_D_ (^2^R_L_)), the competition with the backward reaction (racemization) of [1] is a necessary condition for SMSB. The final composition corresponds to a thermodynamically controlled final stationary state, *i.e.* is independent of the initial concentrations for specific reaction rate parameters, which also includes the total chemical mass. First row of graphics: formation from high initial concentrations of the resources A and B. Second row of graphics: initial racemic composition of chiral replicators and initial conversion towards the achiral resources A and B.

Fig. 2 shows a characteristic example of SMSB for the reaction network [1] + [3] (Fig. 1) in a thermodynamic system closed to matter transfer of the chemical species of the hypercycle but open to external reagents (X/Y), that drive selectively the autocatalysis. Such a chemo-energetic driving of a specific reaction is an analog to what takes place in a cell or could have occurred in abiotic compartmentalized scenarios, by means of a reactant exchange through the membrane. This lifts the chemical thermodynamic constraint involving reactions [1] and [3] (Fig. 1). The dynamics of systems such as that shown in Fig. 2 were obtained by numerical integration of the chemical kinetic differential equation set corresponding to the reaction network (see Methods in ESI†). A stationary final state was always observed, and that within a range of certain reaction rate constants, corresponds to a large enantiomeric excess (ee) in the final state. In the simulations shown in Fig. 2 if the corresponding rate constants are significantly lower than those of cross-catalytically aided autocatalysis [3], but higher than those for direct formation [1], then SMSB may also occur (see Fig. S3 in ESI†). SMSB also takes place when the resources for the replicators are simplified to be one and the same. It is worth noting that for the same system as Fig. 1, and when the hypercycle stoichiometry is transcribed in terms of a sequence of mono- and bimolecular reactions, assuming Michaelis–Menten-like steps, then SMSB can also occur (see the Fig. S4 in ESI†).

The reverse reaction of the direct transformation [1] that leads to the racemization of the chiral replicator in a thermodynamically isolated system is a necessary condition for SMSB in the thermodynamic system in Fig. 2. However, this direct formation of the replicators [1] must be not only slower than [3] but also endergonic or only very slightly exergonic. The role of this backward reaction towards the achiral resource is to destabilize the racemic state with respect to the two energetically degenerate chiral states. A system such as that in Fig. 2 was studied by algebraic stability analysis (see ESI†). The principal conclusions of this analysis are: (a) in the case of the reaction network [1] + [A + R_D_(2R_L_) + X ⇆ 2R_D_(2R_L_) + Y] the final state is always a racemic stationary state; (b) in the case of a chemo-driven second-order autocatalysis (cubic non-linearity: A + 2R_D_(2R_L_) + X ⇆ 3R_D_(3R_L_) + Y) the backward reaction pathway of the direct transformation [1] is a necessary condition for SMSB, as in the case of hypercycle autocatalysis. Such similar behavior between second order autocatalysis and hypercyclic autocatalysis is in fact the main characteristic of the hypercycle model, namely, to convert a first-order autocatalysis into a second order one thus determining selectivity effects affecting the entire set of cross-catalytically coupled replicators.

The dynamics of chiral hypercycles were also studied in other thermodynamic architectures not driven by external reagents (*e.g.*, open flow) but which also prevent the system from equilibrating with its surroundings.

### Inhomogeneous temperature distribution

Hypercyclic replicators [3] in a system closed to matter transfer but subjected to a temperature gradient can also lead to SMSB. This outcome was simulated for a system of homogeneous matter distribution formed by two compartments held at different temperatures and exchanging solution in perfectly mixed conditions. SMSB occurs in a range of certain reaction rate constants when the equilibrium constants are exergonic in one compartment and endergonic in the other one. In the formation of polymeric replicators from simple chemical resources, exothermic transformations with a negative entropy contribution are to be expected, in such cases *K*_eq_ < 1 and *K*_eq_ > 1 can result in the high and low temperature compartments, respectively. In the ESI,† an example (Fig. S5†) of this is given that could be taken as a reasonable working assumption for the temperature differences present in a deep ocean hydrothermal vent scenario, where the liquid state of the medium is conserved over a wide temperature range, with a higher temperature but one lower than that of the decomposition of organic compounds. It is worth noting that in such a system, in contrast with one driven by an external reagent, the direct replicator reaction [1] is not necessary for SMSB. This is also the case for the open flow reactor systems discussed in the following section.

### Open flow reactors

**Fig. 3:**
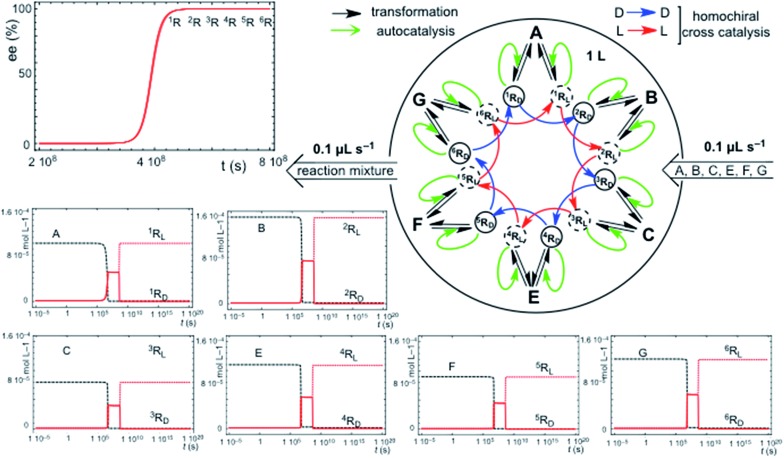
SMSB in an open flow reactor (1 L) for six homochiral cross-catalyzed enantioselective replicators fed by achiral resources. The ee output value (given as the L enantiomeric excess value) is that of full homochirality. Reaction rate constants (*k*_iR_/*k*_–iR_): ^1^R: 1 × 10^4^/10; ^2^R: 1 × 10^3^/0.5; ^3^R: 2 × 10^3^/0.1; ^4^R: 1 × 10^3^/0.1; ^5^R: 2 × 10^3^/0.1; ^6^R: 1 × 10^3^/0.2. Initial resource concentrations in the reactor and in the constant input volume (0.1 μL s^–1^): [A]_o_ = 1 × 10^–4^ mol L^–1^; [B]_o_ = 1.5 × 10^–4^ mol L^–1^; [C]_o_ = 8 × 10^–5^ mol L^–1^; [E]_o_ = 1.5 × 10^–4^ mol L^–1^; [F]_o_ = 9 × 10^–5^ mol L^–1^; [G]_o_ = 1.2 × 10^–4^ mol L^–1^. Initial replicator concentrations in the reactor were 1 × 10^–6^ mol L^–1^ for all the replicator enantiomers: the initial chiral fluctuation was simulated by an additional concentration of 1 × 10^–23^ mol L^–1^ in any one of the enantiomers.

Chiral hypercycles in an open flow reactor may also lead to SMSB. Fig. 3 displays a simulation for six cross-catalytically coupled replicators where the cooperative effect of mutual catalysis increases with the number of coupled replicators, yielding a final homochiral composition (ee = 100% value calculated to 30 digits precision). Clearly, SMSB also occurs in the case of a common shared achiral resource for all the replicators. The example of Fig. 3 does not include the direct transformation reactions [1], *i.e.* the reactor achieves stationary replicator concentrations when the replicators are already present, as well as for the case of low concentration values for the initial conditions. SMSB may also occur in the presence of the direct replicator reaction [1] in the reaction network and without initial concentrations of replicators in the reactor or in the input solution (for an example see Fig. S6, in the ESI†).

A significant result is that in such an open system, the hypercycle composed of only two replicators (see Fig. S7 in ESI†) yields already the same final homochiral composition as the six-replicator network of Fig. 3. The point is that, in abiotic scenarios, if the more simple hypercycle replicator can be achieved then SMSB may occur.

Primordial abiotic scenarios for the formation of replicator hypercycles must imply the presence of the direct formation reaction, but the number of coupled autocatalytic replicators would be small, therefore, the final ee is high, but is not homochiral. Subsequently, the evolution of the catalytic functionalities leading to cross-catalysis for an increasing number of coupled replicators would lead to a homochiral ee value (100%).

The principal trend of life is not only the presence of a common chiral sign (homochirality), but the resilience to racemization of systems supporting continuous chiral enantioselective processes transferring chirality. This can only be supported on the basis of reaction networks leading to SMSB. Notice that any small error in chirality transfer during an elementary reaction will be additive, and a decrease of the ee value would occur in the absence of SMSB despite the high enantioselectivity of enzyme-catalyzed processes. When death occurs, such a system cannot be maintained and racemization begins. Therefore, the signature of life would not only be homochirality, but also the resistance to racemization. Notice that the example in Fig. 3 provides a reasonable reductionist speculation for biological homochirality, because the hypercyclic replicators not only support the emergence of life, but also how homochirality could emerge and be preserved in subsequent evolutive processes.

### Homo- *vs.* heterochiral cross-catalysis

**Fig. 4:**
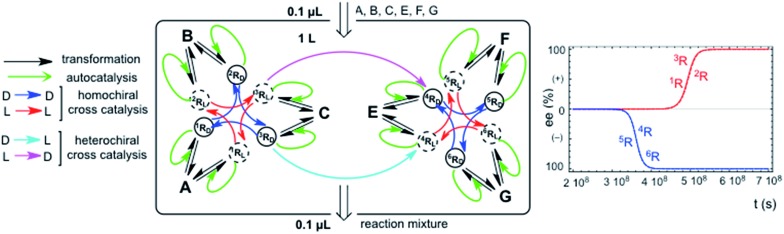
SMSB in an open flow reactor of the reaction network of two homochiral aided hypercycles both being connected by an additional heterochiral cross-catalysis. A chiral fluctuation in any of the replicators leads to 100% homochirality (ee value given as the excess of the L enantiomer). Reaction parameters *k*_iR_/*k*_–iR_. Homochiral catalysis: ^1^R (by ^3^R) 10000/10, ^2^R (by ^1^R) 1000/0.5, ^3^R (by ^2^R) 200/0.1, ^4^R (by ^6^R) 1103/0.3, ^5^R (by ^4^R) 2000/0.2, ^6^R (by ^5^R) 1000/0.2. Heterochiral catalysis: ^4^R (by ^3^R) 100/0.03. Initial resource concentrations in the reactor and in the constant input volume (0.1 μL s^–1^): [A]_o_ = 1 × 10^–4^ mol L^–1^; [B]_o_ = 1.5 × 10^–4^ mol L^–1^; [C]_o_ = 8 × 10^–5^ mol L^–1^; [E]_o_ = 1.5 × 10^–4^ mol L^–1^; [F]_o_ = 9 × 10^–5^ mol L^–1^; [G]_o_ = 1.2 × 10^–4^ mol L^–1^. Initial replicator concentrations in the reactor were 1 × 10^–6^ mol L^–1^ for all enantiomers (a chiral fluctuation was simulated by an additional concentration of 1 × 10^–23^ mol L^–1^ in any of the enantiomers: L in this figure) of the hypercycle ^1^R → ^2^R → ^3^R → ^1^R. Final stationary state (mol L^–1^): [^1^R_L_]_→∞_ = 9.98 × 10^–5^; [^2^R_L_]_→∞_ = 1.49 × 10^–4^; [^3^R_L_]_→∞_ = 7.76 × 10^–5^; [^4^R_D_]_→∞_ = 3 × 10–4; [^5^R_D_]_→∞_ = 9 × 10^–5^; [^6^R_D_]_→∞_ = 1.2 × 10^–4^. The final concentrations of the non-selected enantiomers were <1 × 10^–639^ mol L^–1^.

The chemical assumption implicit in the simple hypercyclic replicator systems discussed here is that the transformations correspond to a reaction type for a family of compounds which possess the same reactant functional group, but different carbon atom backbones: the differences between the reaction rate constants would be due to the effect of the backbone structure on the reaction rate of the functional group. In this respect, homochiral cross-catalysis is the more reasonable chemical assumption, because enantioselective autocatalysis is in fact a homochiral catalysis, and therefore, a catalytic aid for the same type of reaction in the same structural family of chiral replicators should exhibit the same chiral configuration. This means homochirality between autocatalysis and cross-catalysis. However, when the cross-catalysis operates between replicators involving different types of chemical reactions and families of replicators, then the terms homo- and heterochiral cross-catalysis between the replicator families are no longer meaningful. What is important is if the enantiomer acting in one replicator family is the same or the opposite of that acting in the cross-catalysis between the two replicator families. Fig. 4 shows an example of this. This naturally raises speculations on the correlation of the relative chiral configuration of asymmetric centers between nucleic acids and proteins. Our results suggest that the opposite enantiomer should be acting in the cross-catalysis between both replicator families of that acting inside each individual hypercycle family.

### Stochastic *vs.* deterministic final chiral sign: a primordial example of Darwinian chemical selection

**Fig. 5:**
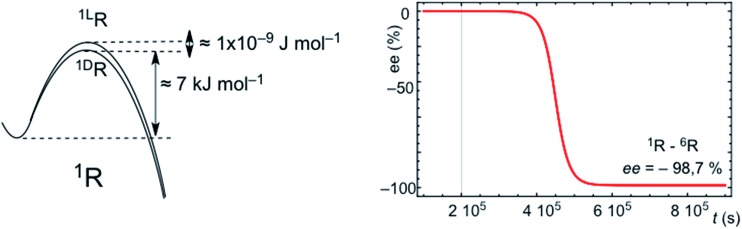
Simulation of the effect of an extremely feeble asymmetric induction converting the final stochastic chiral sign into a deterministic one. The same conditions, system and reaction parameters as those of Fig. S6 are used,† but starting from strictly racemic initial conditions and assuming a very weak chiral induction on the hypercyclic autocatalysis of ^1^R: (*k*_D_ = 1 × 10^5^ + 1 × 10^–9^; *k*_–D_ = 1 × 10^1^ + 1 × 10^–9^ and *k*_L_ = 1 × 10^5^; *k*_–L_ = 1 × 10^1^).

SMSB processes yield a stochastic distribution of chiral signs between successive experiments or outcomes. This means that for a large number of experiments, or in a scenario of a large number of independent working reactors, as in the abiotic scenario of hydrothermal vents in the Hadean Ocean, the average over all the individual stochastic outputs must be the racemic mixture. However, a characteristic of SMSB bifurcations is that a very weak external chiral polarization can select one of the two degenerate chiral branches.^20,21^ Therefore, any weak chiral induction on the enantiomeric transition states, sufficiently weak so that it has no chemical relevance for common asymmetric synthesis, selects one of the two SMSB bifurcation branches deterministically for one of the two chiral signs. There are recent dramatic experimental examples of this for the Soai reaction.^13^ Thus, for example Fig. 5 shows a simulation for the deterministic effect of an extremely weak chiral polarization that converts the enantiomeric transition states into diastereomeric. In summary, any chiral force present in the medium, and which remains active over a relatively long time period, would determine the final chiral sign of all the SMSB reactors placed within this medium. Note that the most common chiral polarization in an abiotic scenario would be a small bias from the racemic composition in the mixture of chiral building blocks constituting the resource pool for replicator synthesis. In this regard, it is now widely accepted that primordial organic compounds were introduced to the early Earth by planetesimal objects,^22^ and experimental evidence strongly suggests that a net chirality of these compounds was present to some ee value during the stages of chemical evolution on Earth.^23^ The effect of permanent and natural chiral forces, acting over vast spatial regions, would also suffice to convert the stochastic final chiral signs of SMSB processes into a coherent deterministic one. All these chiral polarizations would act to transform the stochastic SMSB outcomes into deterministic ones. Homochirality would arise from competitive and cooperative chiral recognition events and not at as singular nor as an accidental event. In summary, the emergence of homochirality in hypercyclic replicators would be an expression of Darwinian evolution at the abiotic chemical level: in a SMSB process only the fittest of both enantiomers will survive. The environment, through the action of spatially extended chiral physical forces, selects the chiral sign. Whether biological homochirality is an accident that occurred during chemical evolution is a separate question that lies beyond the scope of this communication. However, in our opinion only chirality, by virtue of informational entropic reasons, can drive the evolution of chemical systems towards more specific and complex catalytic functionalities.

## Conclusions

Enantioselective hypercycles enable quadratic (first-order) autocatalysis to achieve the enantioselective behavior of cubic (second-order) autocatalysis and therefore may lead to SMSB (a chiral final stationary state instead of a racemic one) for specific reaction rate constants in systems of thermodynamic architectures impeding the system from equilibrating with its surroundings. The significance of such a SMSB reaction network is that it does not imply heterochiral inhibiting reactions such as those of the Frank-like models and, as a consequence, the emergence of biological homochirality could already be included, both theoretically and experimentally, in the current models of the selection and evolution of biological replicators. These results suggest an abiotic scenario of a simultaneous emergence of biological homochirality during the formation of replicator networks with catalytic activity (*e.g.*, in the RNA-world). Furthermore, such a hypothesis also implies that the actual chiral machinery present in extant living beings, capable of transferring chirality and which is highly resistant to racemization, is a complex SMSB network which is a descendant of the primordial ones.
