# Supplementary material for: Spontaneous mirror symmetry breaking in heterocatalytically coupled enantioselective replicators
Source: Chem Sci. 2016 Sep 13;8(1):763–9. doi: 10.1039/c6sc02446g (PMC5299794; doi:10.1039/c6sc02446g)
Supplement: Supplementary file 1 [file SC-008-C6SC02446G-s001.pdf]

Electronic Supplementary Material (ESI) for Chemical Science.
This journal is © The Royal Society of Chemistry 2016

Supplementary Information to

Spontaneous mirror symmetry breaking in heterocatalytically coupled enantioselective replicators

Josep M. Ribó,* Joaquim Crusats,[1,2] Zoubir El-Hachemi,[1,2] Albert Moyano,[1] David Hochberg*

Methods

Simulations were performed by numerical integration of the differential rate equations according to rate-equation theory as applied to chemical kinetics (mean field assumption). The concentration units are mol L^{-1} and the different rate constants have the rate values in units of mol s^{-1}. Numerical integration was performed with the Mathematica® program package. For a set of parameters corresponding to the system at, or very near to, the bifurcation point the numerical integration is highly sensitive to minute differences between the reaction parameters, so that the inherent numerical noise of the calculations suffices to bifurcate the system towards a chiral outcome or made insensitive to SMSB. In our simulations we have suppressed this computational noise, arising from round-off errors, by setting a high numerical precision of the input parameters (100 significant decimal digits and exact number representation of the reaction rates and the initial concentration values (for example "$1 + 1 \cdot x\ 10^{-2}$" instead of "1.01" or "$1. + 1\ x\ 10^{-10}$" or "$1 + 1.\ x\ 10^{-2}$"). Integration methods of "StiffnessSwitching" and "WorkingPrecision" of up to 50 were used in the present calculations.

The *ee* is expressed in % by 100 x (([L] – [D])/ (([L] + [D])). The fluctuations of chirality able to take the system out from the racemic branch were simulated by using an initial ee of products/catalysts lower than that expected from the statistical fluctuations about the ideal racemic composition, i.e an initial ee (%) < 67.43 x ($N^{-0.5}$), where N is the number of chiral molecule.[S1] In the examples shown here the initial *ee* was an order of magnitude lower than that expected from the chiral statistical fluctuation. The numerical integration was run between 0 s to 1 x 10^{20} s. This limit of time, three orders of magnitude larger than the age of the universe, allows us to estimate the concentration value of the stationary state. In the simulations presented here SMSB occur between one day (of chemical significance in potential applied synthesis) and a few decades of years. The effect of an external chiral permanent perturbation of the transition states can be simulated taking $k_{iD} \neq k_{iL}$. In this case values as low as k_{iD}/k_{iL} = 1 x 10^{-9} (transition free energy differences at room temperature in the order of mJ mol L^{-1}) suffice to drive the systems at SMSB conditions from the racemic to the chiral state.

The search of the reaction parameters leading to SMSB was, in the case of the two-replicator system of Figs. 2, S3 and S4, previously estimated from the algebraic adimensional analysis conclusions (Fig. S2.). However, for the compartmentalized system at two temperatures and for hypercycle composed by several replicators the parameter region for SMSB was searched by trial and error of numerical simulations. A complex behavior was observed, for example in the chemical mass, that in simple Frank like systems represents a value below or above which SMSB can be obtained. In the systems studied here, we found regions of chiral solutions bounded by two critical values of the total chemical mass.

Stability studies

1 Single Chiral Replicator: Quadratic autocatalysis

We consider direct production of the replicator enantiomers (R_L and R_D) from an achiral source A:

$$A \underset{k_0}{\overset{k_{-0}}{\rightleftharpoons}} R_L(R_D) \qquad [S1]$$

and *quadratic* autocatalysis driven by constant concentration external reagents X,Y:

$$A + R_L(R_D) + X \underset{k_R}{\overset{k_{-R}}{\rightleftharpoons}} 2\,R_L(2\,R_D) + Y \qquad [S2]$$

such that the number of "internal" molecules A+R_L+R_D is conserved. These correspond to the transformations [1] and [2] of Scheme 1 in the main text. The temporal T and spatial V physical dimensions of the reaction rate constants (indicated here by the brackets)

$$[k_0] = [k_{-0}] = T^{-1}, \qquad [S3]$$

and

$$[k_R] = [k_{-R}] = T^{-1}V, \qquad [S4]$$

allow us to express the associated kinetic rate equations in terms of dimensionless rates and concentrations [1, 2]. Changing variables to $\chi = R_D+R_L$, [S1,S2] then imply [see for example refs. S2 and S3 for more details].

$$\frac{d\chi}{d\tau} = 2A + (A-u)\chi - \frac{g}{2}(\chi^2 + y^2), \qquad [S5]$$

$$\frac{dy}{d\tau} = y(A - u - g\chi), \qquad [S6]$$

where $\tau = k_0 t$ is dimensionless time, $(A,\chi,y) = \frac{k_R}{k_0}([A],[\chi],[y])$ the dimensionless concentrations, and

$$A = C - \chi, \qquad u = \frac{k_{-0}}{k_0}, \qquad g = \frac{[Y]k_{-R}}{[X]k_R}. \qquad [S7]$$

Equations (S5,S6) admit one racemic and two chiral stationary solutions:

$$y = 0, \qquad \chi = \frac{C - 2 - u + \sqrt{C^2 + C(4 + 4g - 2u) + (2+u)^2}}{2+g}, \qquad [S8]$$

$$y = \pm\frac{\sqrt{C^2 g + 2Cg(2+2g-u) + u(4 + g(4+u))}}{\sqrt{g(1+g)^2}}, \qquad \chi = \frac{C-u}{1+g}. \qquad [S9]$$

To assess dynamic stability, we linearize the rate equations [S5,S6] and consider arbitrary fluctuations around the stationary solutions. Their time dependence is determined by

$$\frac{d}{d\tau}\begin{pmatrix}\delta y\\ \delta\chi\end{pmatrix} = \mathrm{A}\begin{pmatrix}\delta y\\ \delta\chi\end{pmatrix}, \qquad [S10]$$

where

$$\mathbf{A} = \begin{pmatrix} A - u - g\chi & -(g+1)y \\ -gy & A - 2 - u - (g+1)\chi \end{pmatrix}. \qquad [S11]$$

We evaluate the array $\mathbf{A}$ over one of the stationary solutions [S8,S9] (racemic or chiral) and compute the corresponding pair (λ_1, λ_2) of eigenvalues. Doing so for the racemic solution we find:

$$\lambda_1 < 0 \,\&\, \lambda_2 < 0, \qquad \forall C > 0, g > 0, u > 0, \qquad \text{(racemic)} \qquad [S12]$$

whereas for both the chiral solutions we find

$$\lambda_1 < 0 \,\&\, \lambda_2 > 0, \qquad \forall C > 0, g > 0, u > 0, \qquad \text{(chiral)}. \qquad [S13]$$

This establishes that quadratic autocatalysis together with direct production yields the racemic solution as the unique final stable state, despite the fact that the autocatalysis is driven by external reagents.

2 Single Chiral Replicator: Cubic autocatalysis

We consider direct production of the replicator enantiomers (R_L and R_D) from an achiral source A:

$$A \underset{k0}{\overset{k_{-0}}{\rightleftharpoons}} R_L(R_D) \qquad [S14]$$

and *quadratic* autocatalysis driven by constant concentration external reagents X,Y:

$$A + 2\,R_L(R_D) + X \underset{k_R}{\overset{k_{-R}}{\rightleftharpoons}} 3\,R_L(R_D) + Y \qquad [S15]$$

such that the number of "internal" molecules A+R_L+R_D is conserved. These correspond to the transformations [1] and [2] of Scheme 1 in the main text. The temporal T and spatial V physical dimensions of the reaction rate constants (indicated here by the brackets)

$$[k_0] = [k_{-0}] = T^{-1}, \qquad [S1$$

and

$$[k_R] = [k_{-R}] = T^{-1}V^2, \qquad [S17]$$

allow us to express the associated kinetic rate equations in terms of dimensionless rates and concentrations [1, 2]. Changing variables to $\chi = R_D + R_L$, [S14,S15] then imply [see for example refs.[25,26] for more details].

$$\frac{d\chi}{d\tau} = 2A - u\chi + \frac{A}{4}((\chi+y)^2 + (\chi-y)^2) - \frac{g}{8}((\chi+y)^3 + (\chi-y)^3), \qquad [S18]$$

$$\frac{dy}{d\tau} = -uy + \frac{A}{4}((\chi+y)^2 - (\chi-y)^2) - \frac{g}{8}((\chi+y)^3 - (\chi-y)^3), \qquad [S19]$$

where $\tau = k_0 t$ is dimensionless time and

$$A = C - \chi, \qquad u = \frac{k_{-0}}{k_0}, \qquad g = \frac{[Y]k_{-R}}{[X]k_R}. \qquad [S20]$$

The *dimensionless* concentrations appearing in [S18,S19] are obtained by rescaling their dimensionfull counterparts as follows $(A, C, \chi, y) = (\frac{k_R}{k_0})^{1/2}([A],[C],[\chi],[y])$.

2.1 No racemization $\Rightarrow$ no SMSB

If we *omit* the forward/reverse direct production steps [S14], then proceed with the non-dimensionalization, the stationary solution of the resultant [S18] is $\chi = \frac{2C}{2+g} > 0$ for y = 0 [and for which [S19] holds identically). The corresponding eigenvalues for time-depende fluctuations $\delta\chi(\tau)$ and $\delta y(\tau)$ around this racemic state are

$$\lambda_\chi = -\frac{1}{2}\chi^2 + A\chi - \frac{3g}{4}\chi^2, \qquad [S21]$$

$$\lambda_y = +A\chi - \frac{3g}{4}\chi^2. \qquad [S22]$$

Both $\lambda_\chi < 0$, λ_y are negative when evaluated on the above solution for χ, implying the stability of the mirror symmetric state. There is thus no SMSB in the absence of the direct production steps, regardless of the values of g and the total system concentration C.

2.2 Racemization: Phase space for SMSB

When the direct production steps *are* reinstated, when $y = 0$ we must solve the cubic equation

$$-\frac{g}{4}\chi^3 + \frac{1}{2}(C-\chi)\chi - u\chi + 2(C-\chi) = 0 \qquad [S23]$$

for the positive solutions $\chi > 0$.

The pair of eigenvalues are given by

$$\lambda_\chi = -2 - u - \frac{1}{2}\chi^2 + A\chi - \frac{3g}{4}\chi^2, \qquad [S24]$$

$$\lambda_y = -u + A\chi - \frac{3g}{4}\chi^2. \qquad [S25]$$

Now λ_y can be positive on the positive solutions to [S23]. The phase space for SMSB is described by the three parameters *u*, *g*, *C* defined in [S20]. A representative region where mirror symmetry is *broken* ($\lambda_y > 0$) is depicted in the phase diagram Fig. S1.

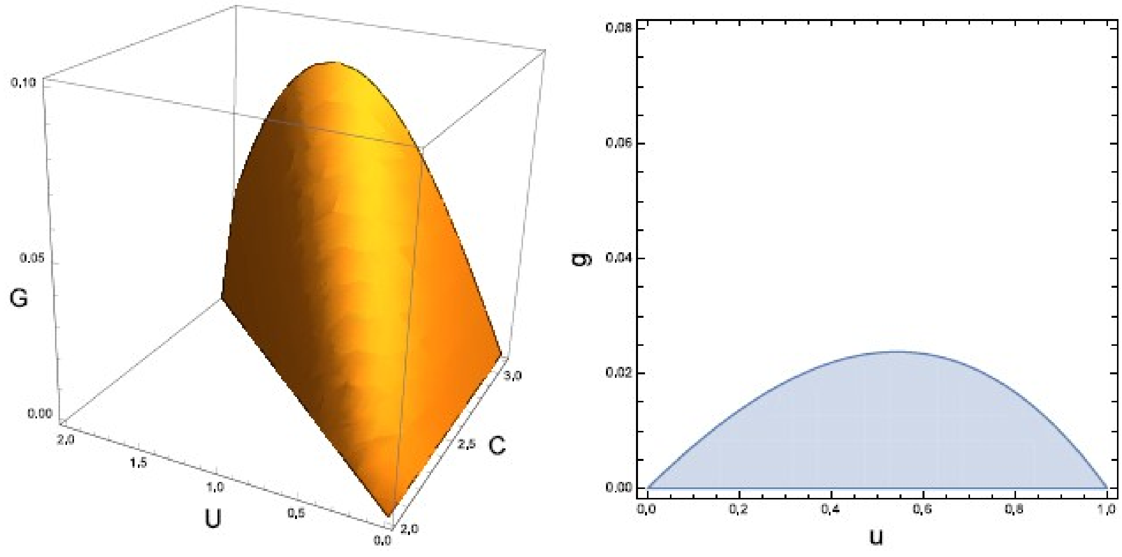

Figure S1. Effect of varying total system concentration C, the ratio u of the rates of inverse to direct production and the ratio g of the rates of inverse to forward cubic autocatalysis (driven by external reagents). Left: the three dimensional region represents points (colored) for which the racemic state $y = 0$ is unstable to small perturbations ($\lambda y > 0$) and hence where SMSB results. Right: a transverse “slice" of this region at C =2.5 indicting the nonlinear dependence of g on u and the maximum value of the former. Left: That maximum increases with C. For a given system concentration $C > C_{min}$, there is a finite range in both u and g for which SMSB occurs. Now u ♑ 0 corresponds a rapid racemization or direct production rate with respect to its inverse: $k_0 >> k_{-0}$. The finite upper bound on u corresponds to the opposite situation, namely when $k_{-0} << k_0$ But the forward rate of direct production k_0 cannot be too small, otherwise there is no SMSB. The limit g ✂ 0 corresponds to relative fast rates of the forward to reverse (driven) cubic catalysis. For a given u, there is a finite upper bound on g indicating *minimum* values of (driven) forward catalysis needed to set off SMSB. Note this range of minimal rates of forward (driven) catalysis is *maximized* for intermediate values of u (right hand side Fig.S1.). Increasing C widens both the range in u and the maximum value of g for which SMSB can occur.

3 The Two Replicator Hypercycle: replicators aided by homochiral cross-catalysis

We start from the reactions [1] and [3] of Scheme 1 of the main article for two replicators and here consider a single achiral source A for both in the interest of mathematical simplicity. Introduce the dynamical variables [1]

$$\chi^{(1)} = R_L^{(1)} + R_D^{(1)}, \qquad y^{(1)} = R_L^{(1)} - R_D^{(1)}, \tag{S26}$$

$$\chi^{(2)} = R_L^{(2)} + R_D^{(2)}, \qquad y^{(2)} = R_L^{(2)} - R_D^{(2)}. \tag{S27}$$

From the temporal T and spatial V physical dimensions of the reaction rates

$$[k_{01}] = [k_{-01}] = [k_{02}] = [k_{-02}] = T^{-1}, \tag{S28}$$

and

$$[k_{HH1}] = [k_{-HH1}] = [k_{HH2}] = [k_{-HH2}] = T^{-1}V^{1}, \tag{S29}$$

we express the kinetic rate equations in dimensionless form [1, 2]

$$\frac{d\chi^{(1)}}{d\tau_1} = 2A - u\chi^{(1)} + \frac{A}{4}((\chi^{(1)} + y^{(1)})(\chi^{(2)} + y^{(2)}) + (\chi^{(1)} - y^{(1)})(\chi^{(2)} - y^{(2)}))$$

$$-\frac{g}{8}((\chi^{(1)} + y^{(1)})^2(\chi^{(2)} + y^{(2)}) + (\chi^{(1)} - y^{(1)})^2(\chi^{(2)} - y^{(2)})),$$

$$\frac{dy^{(1)}}{d\tau_1} = -uy^{(1)} + \frac{A}{4}((\chi^{(1)} + y^{(1)})(\chi^{(2)} + y^{(2)}) - (\chi^{(1)} - y^{(1)})(\chi^{(2)} - y^{(2)})) \tag{S30}$$

$$-\frac{g}{8}((\chi^{(1)} + y^{(1)})^2(\chi^{(2)} + y^{(2)}) - (\chi^{(1)} - y^{(1)})^2(\chi^{(2)} - y^{(2)})). \tag{S31}$$

and

$$\frac{d\chi^{(2)}}{d\tau_2} = 2A - v\chi^{(2)} + \frac{sA}{4}((\chi^{(1)} + y^{(1)})(\chi^{(2)} + y^{(2)}) + (\chi^{(1)} - y^{(1)})(\chi^{(2)} - y^{(2)}))$$

$$-\frac{h}{8}((\chi^{(2)} + y^{(2)})^2(\chi^{(1)} + y^{(1)}) + (\chi^{(2)} - y^{(2)})^2(\chi^{(1)} - y^{(1)})), \tag{S32}$$

$$\frac{dy^{(2)}}{d\tau_2} = -vy^{(2)} + \frac{sA}{4}((\chi^{(1)} + y^{(1)})(\chi^{(2)} + y^{(2)}) - (\chi^{(1)} - y^{(1)})(\chi^{(2)} - y^{(2)})).$$

$$-\frac{h}{8}((\chi^{(2)} + y^{(2)})^2(\chi^{(1)} + y^{(1)}) + (\chi^{(2)} - y^{(2)})^2(\chi^{(1)} - y^{(1)})), \tag{S33}$$

where dimensionless time-parameters are $\tau = k_{0j}t$ for $j = 1,2$, and

$$u = \frac{k_{-01}}{k_{01}}, \qquad v = \frac{k_{-02}}{k_{02}}, \qquad g = (\frac{[Y]k_{-HH1}}{[X]k_{HH1}}), \qquad h = (\frac{[Y]k_{-HH2}}{[X]k_{HH1}})\frac{k_{01}}{k_{02}}, \qquad s = \frac{k_{HH2}}{k_{HH1}}\frac{k_{01}}{k_{02}}. \tag{S34}$$

All the concentrations in the above rate equations are dimensionless. The relation between the dimensionless and dimensionfull concentrations (e.g., [A]) is given by

$$A = (\frac{k_{HH1}}{k_{01}})^{1/2}[A]. \tag{S35}$$

The total system concentration C is conserved:

$$A + \chi^{(1)} + \chi^{(2)} = C. \tag{S36}$$

The dynamics is thus described by the six independent dimensionless parameters in [S34,S36].

We seek stationary solutions $\chi(1)$, $\chi^{(2)}$ corresponding to the *global racemic* state $y^{(1)} = y^{(2)} = 0$. In this configuration, the fluctuations in the net chiral mass in each species $\delta\chi^{(1)}$, $\delta\chi^{(2)}$ *decouple* from the chiral fluctuations about the racemic $\delta y^{(1)}, \delta y^{(2)}$ and so the 4x4 Jacobian matrix decomposes into block-diagonal form with the two 2x2 sub-blocks:

$$A = \begin{pmatrix} -2 - u - (g+1)\frac{\chi^{(1)}\chi^{(2)}}{2} + \frac{1}{2}(c - \chi^{(1)} - \chi^{(2)})\chi^{(2)} & -2 - \frac{g(\chi^{(1)})^2}{4} + \frac{1}{2}(c - \chi^{(1)} - \chi^{(2)})\chi^{(1)} - \frac{\chi^{(1)}\chi^{(2)}}{2} \\ -2 - \frac{h(\chi^{(2)})^2}{4} + \frac{s}{2}(c - \chi^{(1)} - \chi^{(2)})\chi^{(1)} - \frac{s\chi^{(1)}\chi^{(2)}}{2} & -2 - v - (h+s)\frac{\chi^{(1)}\chi^{(2)}}{2} + \frac{s}{2}(c - \chi^{(1)} - \chi^{(2)})\chi^{(1)} \end{pmatrix} \quad \text{[S37]}$$

and

$$B = \begin{pmatrix} -u - \frac{g\chi^{(1)}\chi^{(2)}}{2} + \frac{1}{2}(c - \chi^{(1)} - \chi^{(2)})\chi^{(2)} & -\frac{g(\chi^{(1)})^2}{4} + \frac{1}{2}(c - \chi^{(1)} - \chi^{(2)})\chi^{(1)} \\ -\frac{h(\chi^{(2)})^2}{4} + \frac{1}{2}s(c - \chi^{(1)} - \chi^{(2)})\chi^{(2)} & -v - \frac{h\chi^{(1)}\chi^{(2)}}{2} + \frac{1}{2}s(c - \chi^{(1)} - \chi^{(2)})\chi^{(1)} \end{pmatrix}. \quad \text{[S38]}$$

The time dependence of the matter and chiral fluctuations about the global racemic obeys

$$\begin{pmatrix} \frac{d\delta\chi^{(1)}}{d\tau_1} \\ \frac{d\delta\chi^{(2)}}{d\tau_2} \end{pmatrix} = \mathbf{A} \begin{pmatrix} \delta\chi^{(1)} \\ \delta\chi^{(2)} \end{pmatrix} \quad \text{[S39]}$$

and

$$\begin{pmatrix} \frac{d\delta y^{(1)}}{d\tau_1} \\ \frac{d\delta y^{(2)}}{d\tau_2} \end{pmatrix} = \mathbf{B} \begin{pmatrix} \delta y^{(1)} \\ \delta y^{(2)} \end{pmatrix} \quad \text{[S40]}$$

We set $y^{(1)} = y^{(2)} = 0$ in [S31,S33] and solve this pair for the stationary solutions ([S30,S32] hold identically). The ensuing pair of coupled cubic equations can have positive, negative, as well as complex solutions. Only the simultaneous real-valued and positive solutions $\chi^{(1)} > 0$, $\chi^{(2)} = > 0$ correspond to acceptable chemical concentrations. The signs of the eigenvalues of the 2x2 arrays **A** and **B** evaluated over these acceptable solutions indicate whether the racemic solution is stable or not. If *any* one of the (four) eigenvalues is positive, the racemic state is unstable to arbitrary small perturbations which thus trigger the onset of SMSB. A parameter survey leads to the representative phase diagram discussed below.

4 Phase Space for SMSB: $v = u, h = g, s = 1$ and C

The full phase space of this two-replicator system is six-dimensional, nevertheless, we can obtain considerable insight into the roles played by the total system concentration, the racemization and the rates of the (driven) heterocatalyses by specializing to a three dimensional parameter space. To this end, we here set $v = u$, $h = g$, s =1 and then vary u, g, C. Note that h corresponds to the ratio of the rates of inverse to forward catalysis for the species $R^{(2)}$ when s =1, and in this limit is analogous to the parameter g for species $R^{(2)}$:

$$s = 1 \Rightarrow h = \left(\frac{[Y]k_{-\text{[illegible]}}}{[X]k_{\text{[illegible]}}}\right). \qquad [S41]$$

An illustrative region where mirror symmetry is *broken* is depicted in the phase diagram Fig. S2. This is qualitatively similar to the phase portrait for the single replicator case discussed above Fig. S1. A notable quantitative distinction is that the *minimum* value of C required for SMSB or two-replicators has increased with respect to the single replicator case.

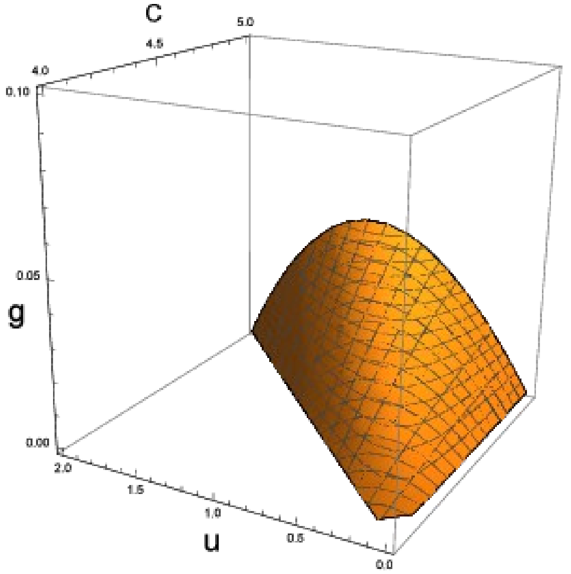

Figure S2. Effect of varying total system concentration C, versus the ratio u = v of the rates of inverse to direct production of both species $R^{(1)}$, $R^{(2)}$ and the ratio $g = h$ of the rates of reverse to forward autocatalysis of both species (driven by the external reagents). Here s =1. The three dimensional figure represents points for which the global racemic state $y^{(1)} = y^{(2)} = 0$ is unstable to small time-dependent perturbations.

References (Supplementary Information)

K. Mislow, *Collect. Czech. Chem. Commun.* 2003, **68**, 849–864.

J. M. Ribo and D. Hochberg, Stability of racemic and chiral steady states in open and closed chemical systems. *Phys. Lett. A* 2008, 373, 111-122.

C. Blanco, J. M. Ribó, J. Crusats, Z. El-Hachemi and D. Hochberg, Mirror symmetry breaking with limited enantioselective autocatalysis and temperature gradients: a stability survey. *Phys. Chem. Chem. Phys.* 2013, **15**, 1546-155.

Figures. S3 – S7: Examples of SMSB of chiral hypercyclic autocatalysis (comments in the main text)

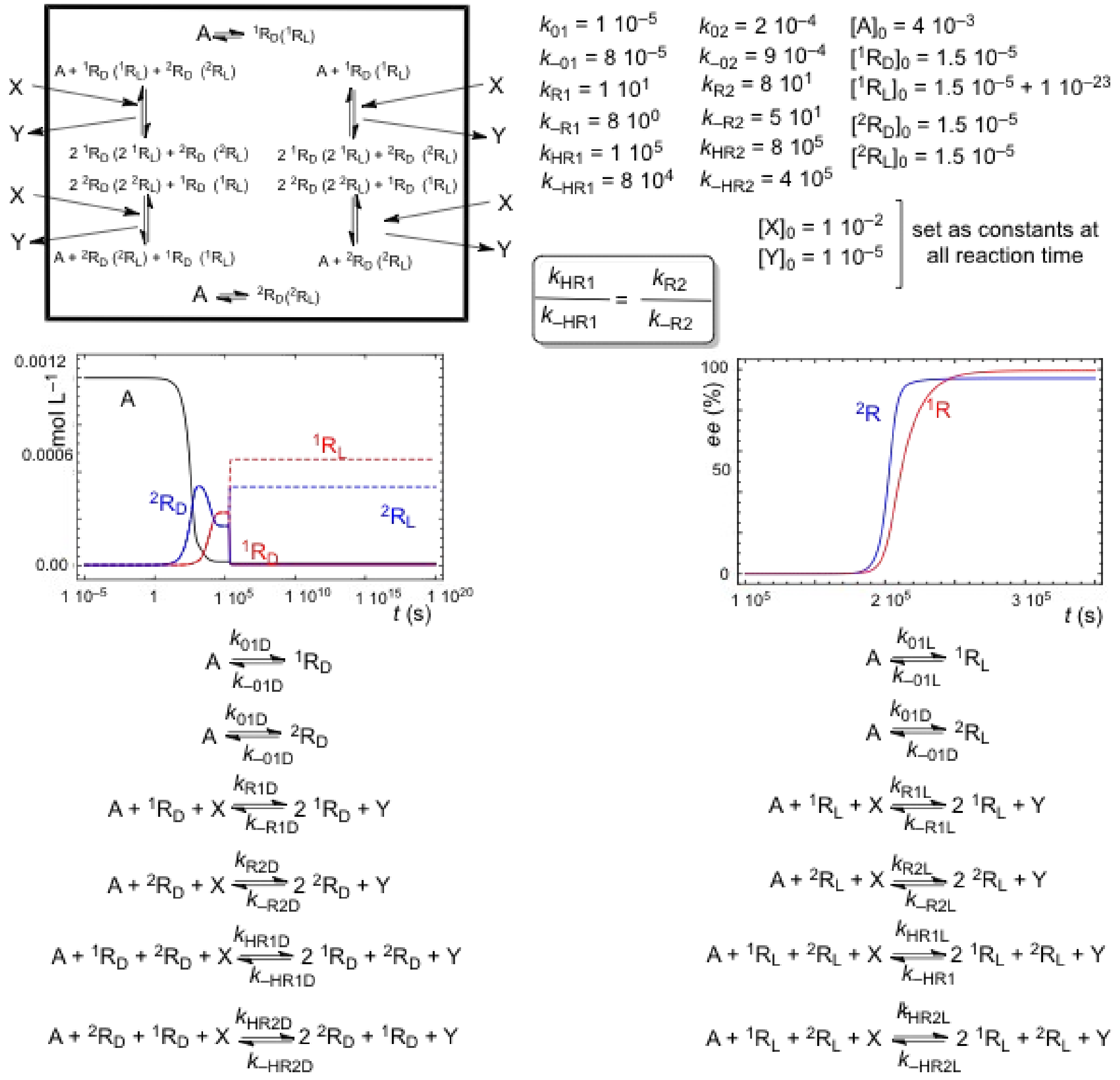

Figure S3. SMSB in the system of Fig. 1 but in a reaction network including also the non-cross catalyzed autocatalysis (2) and the same achiral resource A for the two chiral replicators (^1R_D (^1R_L) and ^2R_D (^2R_L)). The autocatalysis (2) as well as (3) are driven by the reagent X/Y at constant concentrations.

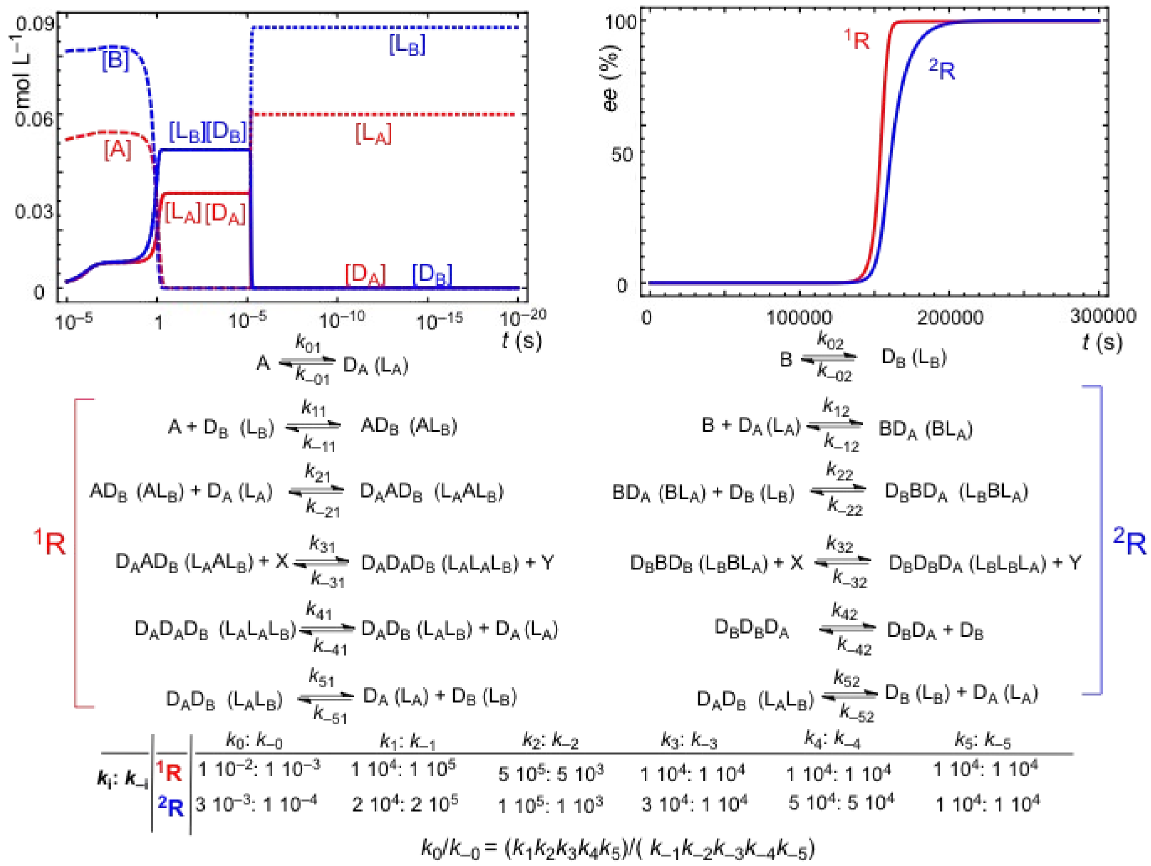

Figure S4. SMSB for a similar reaction network and system as those of Fig. 1 but under reaction mechanisms implying only mono- and bimolecular order reactions. Initial concentrations: $[X]_{constant}$ = 0.01 mol L^{-1}; $[Y]_{constant}$ = 1 x 10^{-6} mol L^{-1}; $[A]_o$ = 0.05 mol L^{-1}; $[B]_o$ = 0.08 mol L^{-1}; The initial concentration of the rest of compounds was 1 mmol L^{-1} except for $[L_A]_o$ = (1 x 10^{-3} + 1 x 10^{-23}) mol L^{-1} (see Methods) The initial concentration of the rest of the species was set to zero. The same final state when starting from a different relationship of initial concentrations, i.e. the final species composition corresponds to a thermodynamically controlled stationary state.

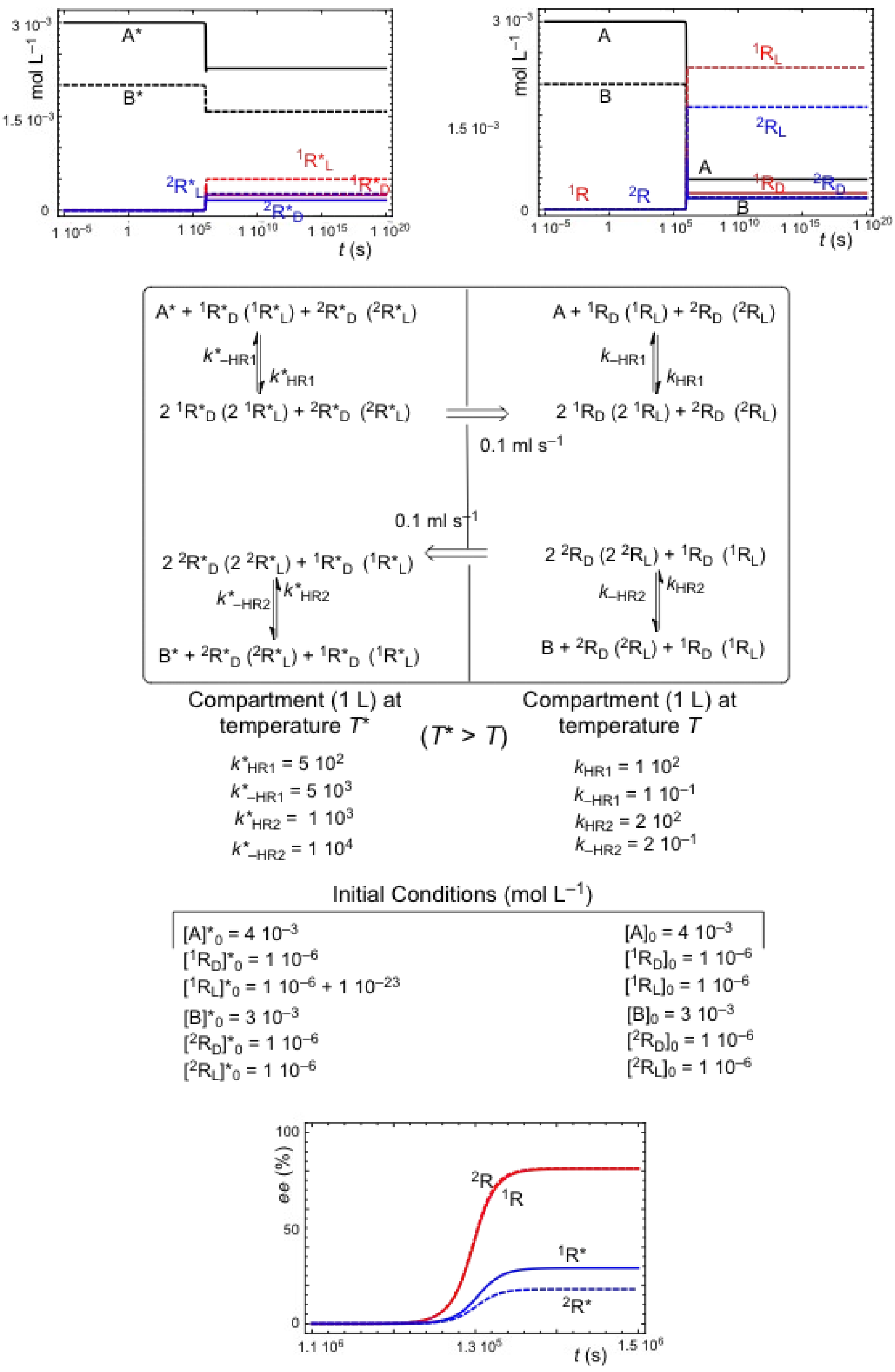

A* + ¹R*D (¹R*L) + ²R*D (²R*L)
k*−HR1
k*HR1
2 ¹R*D (2 ¹R*L) + ²R*D (²R*L)
A + ¹RD (¹RL) + ²RD (²RL)
k−HR1
kHR1
2 ¹RD (2 ¹RL) + ²RD (²RL)
0.1 ml s−1
0.1 ml s−1
2 ²R*D (2 ²R*L) + ¹R*D (¹R*L)
k*−HR2
k*HR2
B* + ²R*D (²R*L) + ¹R*D (¹R*L)
2 ²RD (2 ²RL) + ¹RD (¹RL)
k−HR2
kHR2
B + ²RD (²RL) + ¹RD (¹RL)
Compartment (1 L) at temperature T*
(T* > T)
Compartment (1 L) at temperature T
k*HR1 = 5 10²
k*−HR1 = 5 10³
k*HR2 = 1 10³
k*−HR2 = 1 10⁴
kHR1 = 1 10²
k−HR1 = 1 10−1
kHR2 = 2 10²
k−HR2 = 2 10−1
Initial Conditions (mol L−1)
[A]*0 = 4 10−3
[¹RD]*0 = 1 10−6
[¹RL]*0 = 1 10−6 + 1 10−23
[B]*0 = 3 10−3
[²RD]*0 = 1 10−6
[²RL]*0 = 1 10−6
[A]0 = 4 10−3
[¹RD]0 = 1 10−6
[¹RL]0 = 1 10−6
[B]0 = 3 10−3
[²RD]0 = 1 10−6
[²RL]0 = 1 10−6
ee (%)
t (s)
mol L−1

Figure S5. SMSB of a two replicator chiral hypercycle in a closed system with permanent non-homogeneous temperature distribution: two compartments at different temperature exchanging the same volume of solution. For a more detailed decription of this type of systems see for details *Astrobiology* 2013, **13**, 132-142. The change on equilibrium constants would agree with that expected for temperature differences as those of deep ocean hydrothermal wells (411° K and 275° K) and for an exothermic transformation showing negative entropy, as expected for replicator obtained by polymerizating reactions.

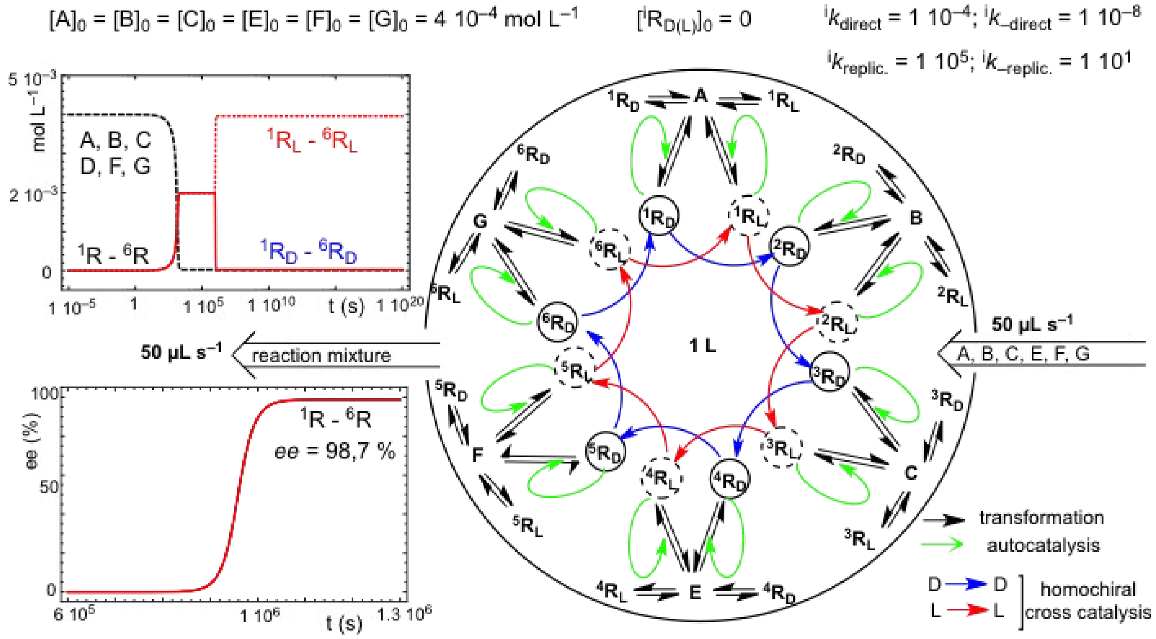

Figure S6. SMSB in a open flow reactor of the reaction network of a six hypercyclic chiral replicator of homochiral cross-catalysis with direct replicator synthesis and no replicator concentration for the initial conditions. A chiral fluctuation in any of the replicators leads to an *ee* near to the 100% value (chiral fluctuation simulated by an initial concentration of 1 x 10^{-25} mol L^{-1} in any of the L-enantiomers).

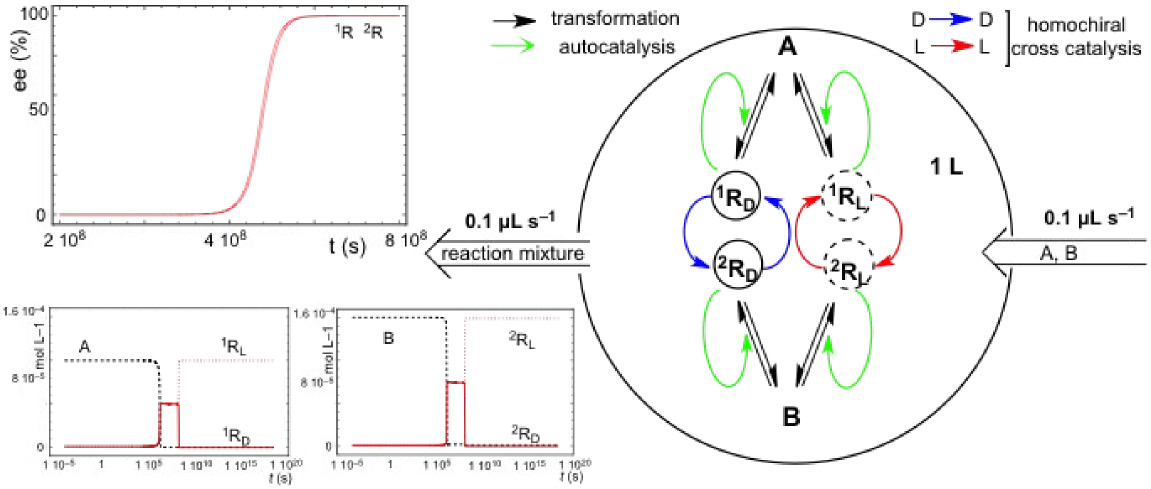

Figure S7. SMSB in an open flow reactor (1 L) for two homochiral cross-catalyzed enantioselective replicators fed by achiral resources (similar to the six-replicators of Fig. 3 in the main text). The *ee* output value (given as L enantiomers excess value) is that of full homochirality. Reaction rate constants (k_{iR}/k_{-iR}): ^{1}R: 1 x 10^4/10; 2R: 1 x 10^3/0.5. Initial resource concentrations in the reactor and in the constant input volume (0.1 µL s–1): $[A]_o$ = 1 x 10^{-4} mol L^{-1}; $[B]_o$ = 1.5 x 10^{-4} mol L^{-1}. Initial replicator concentrations in the reactor were 1 x 10^{-6} mol L–1 and the initial chiral fluctuation was simulated by an additional concentration of $1x10^{-23}$ mol L^{-1} in the L enantiomers.
